# Evaluating palatability in young children: a mini-review of relevant physiology and assessment techniques

**DOI:** 10.3389/fped.2024.1350662

**Published:** 2024-02-08

**Authors:** Haley M. Schluterman, Constance G. Linardos, Teresa Drulia, James D. Marshall, Gregory L. Kearns

**Affiliations:** ^1^ Departments of Medical Education, The Anne Marion Burnett School of Medicine, Texas Christian University, Fort Worth, TX, United States; ^2^ Davies School of Communication Sciences and Disorders, The Harris College of Nursing and Health Sciences, Texas Christian University, Fort Worth, TX, United States; ^3^ Departments of Pediatrics, The Anne Marion Burnett School of Medicine, Texas Christian University, Fort Worth, TX, United States; ^4^ The Divisions of Intensive Care Medicine, Cook Children's Medical Center, Fort Worth, TX, United States; ^5^ The Divisions of Palliative Care, Cook Children's Medical Center, Fort Worth, TX, United States

**Keywords:** medicine, palatability, taste physiology, viscosity, children

## Abstract

The palatability of pediatric pharmaceutical products plays a crucial role of influencing medication compliance. Rejection of unpalatable medications can potentially lead to treatment failure which can have immediate and delayed consequences. With advances in both the food and pharmaceutical industries, the systematic assessment of palatability has gained importance. Various methods such as visual analogue scales, facial hedonic scales, and facial recognition software, have been employed to assess palatability. While proven to be useful, these methods have significant limitations and may not be workable for young children. Despite these advancements, a universally accepted “gold standard” for assessing pediatric mediation palatability, recognized by drug regulatory agencies, is yet to be established.

## Introduction

As recently reviewed by Peng et al. (1), the palatability of pediatric pharmaceutical products for children can adversely influence medication compliance. The rejection of unpalatable pharmaceutical products can negatively impact therapeutic adherence which, in turn can lead to treatment failure which may have both immediate consequences (e.g., in the case of oral liquid antimicrobials, persistence of disease) and potentially, delayed consequences (e.g., fostering antibiotic resistance) (2–4) In a survey of drug administration methods and their relationship to compliance (5), reported that more than 50% of children aged 6 years or less had difficulty swallowing oral drug formulations. Potential contributing factors include the size and shape of a given solid oral drug formulation and in the case of both solids and liquids, factors related to palatability such as taste, flavor, and smell (6–8).

Evaluation of drug palatability and patient acceptance has been done using both *in vitro* and *in vivo* evaluations and has become increasingly important (6). Several methods have been developed and applied for palatability assessment (9) which includes, visual analog scales (10), facial hedonic scales (11), use of an electronic tongue (12) and more recently, the use of facial recognition software (13). Despite these advancements, there appears to be no generally accepted “gold standard” method for palatability assessment in children that has been universally adopted by drug manufacturers or regulatory agencies. In this mini-review, our goal is to provide the reader with a review of the biologic determinants of palatability and also, the existing methods used to assess it in children.

## Background

Palatability is a subjective measure of how pleasant a food or drug substance is to consume. It is influenced by a variety of organoleptic factors which are sensory in nature, individually determined, and most often associated with a specific substance/product. Factors that can influence palatability (Google BARD: https://bard.google.com; accessed 25 October 2023) include the following:

**Sensory Factors** (e.g., taste, texture, temperature, visual appearance)
**Individual Factors** (e.g., age, culture, health status, psychological)
**Product Factors** (e.g., physical form, additives, colorants)

Also, it is possible that the patient environment may also be a contributing factor in the assessment of drug palatability in pediatric patients (e.g., a calm, reassuring environment without distractions being preferrable).

Generally speaking, palatability is purely a subjective experience. What one person finds palatable another person may find unpalatable or objectionable. Additionally, palatability can change over time. For example, a person may develop a taste for a food or drug after repeated exposure or alternatively, taste preference can often change with aging. Finally, taste involves a complex interaction between olfaction, biological perception of flavor and mouthfeel of the substance being tasted (14).

## Relevant biology of human taste

### The role of olfaction in taste perception

The sense of smell is a major determinant of perceived taste of a given substance. Without our sense of smell, our sense of taste is limited to only five distinct sensations: sweet, salty, sour, bitter and “umami” or savory sensation. Consequently, olfaction is relevant as a sense in the acceptance of orally administered medications.

As reviewed by Czarnecki and Fontanini (15), odors have a dual relationship with taste: they can either precede it or accompany it. The former happens when we smell the aroma of the food in front of us through what is known as oronasal smelling. The latter happens when food in the mouth liberates volatile molecules that are carried by air through the pharynx into the nose, a process called retro-nasal smelling. Retronasal olfactory stimulation appears to be bidirectional in that it inextricably links smell and taste perception as overall components of taste sensation.

**Figure 1:**
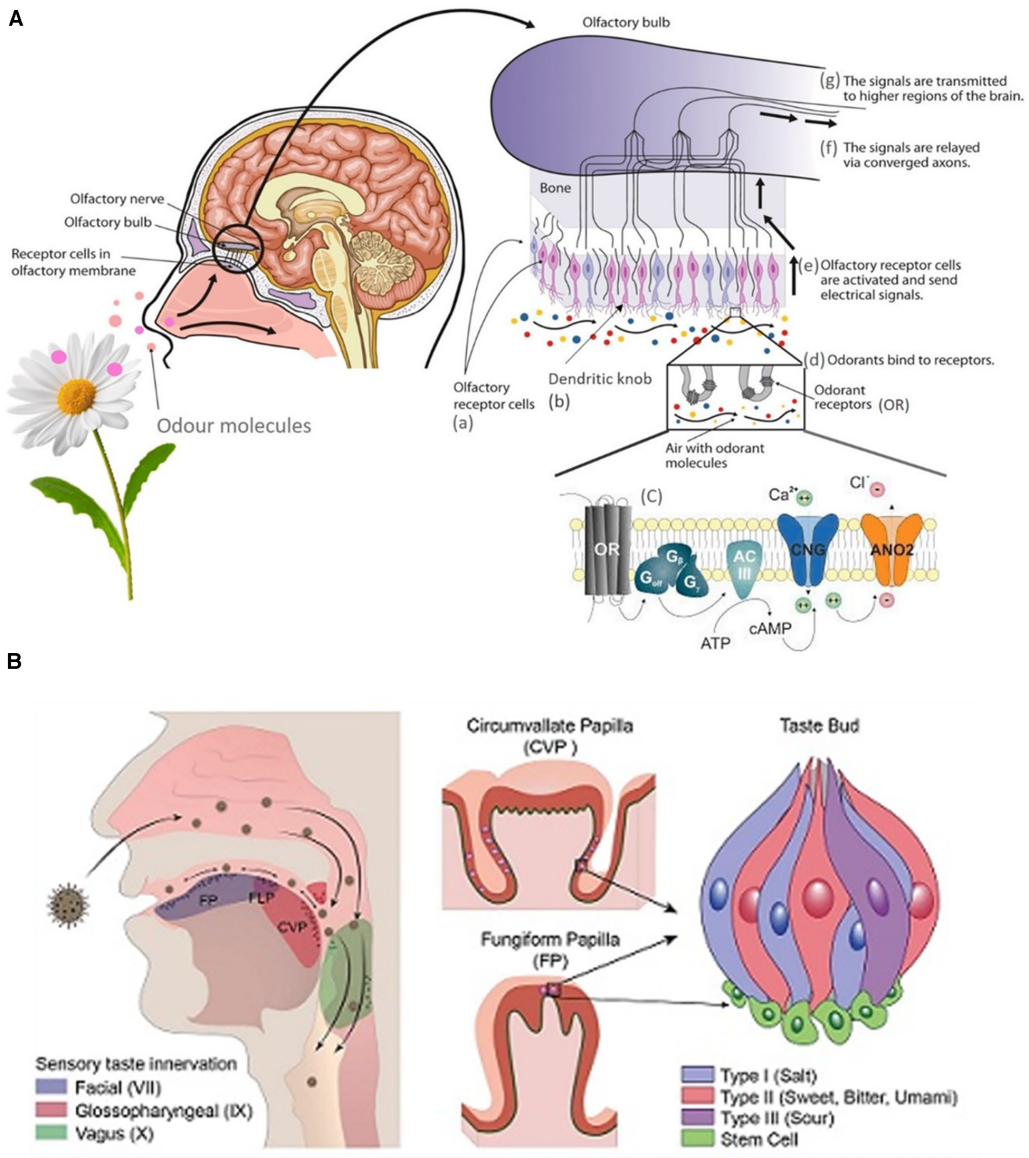
(**A**) Graphical depiction of the biology of olfaction (17). (**B**) Tongue structures and Innervation (18).

The two streams of chemosensory information regulating olfaction and taste have a complex interaction and their interplay occurs well before signals reach the orbitofrontal cortex of the brain (15). Neurons in the gustatory cortex can respond to odors and those in the olfactory cortex can respond to taste (16). The basic biology of olfaction is illustrated in Figure 1A and very recently, “taste coding” at a biomolecular level has been extensively reviewed by Roper (19).

### Determinants of the human sensation of taste

Human taste is characterized by 5 qualities including sweet, sour, salty, bitter, and umami (savory) (20). The main organ of taste is the tongue, which contains sensory endings called papillae. There are two types of papillae: the foliate, fungiform, and circumvallate papillae which house the taste buds, and the filiform papillae that sense pain, temperature, and touch. Additionally, each taste bud is made up of one type of cell (type 1, 2, 3 cells, basal cells, neuronal processes) corresponding to one taste modality. For example, type 1 cells are sensitive to salty tastes, while type 2 detect bitterness. The tongue is innervated by the trigeminal nerve (CN V), glossopharyngeal nerve (CN IX), and the chorda tympani (CN VII), which help send taste information to the gustatory cortex (21). The tongue structures and their sensory innervation is illustrated in Figure 1B (18).

The sweet receptor is composed of two proteins (T1R2 and T1R3) that are encoded by the associated genes TAS1R2 and TAS1R3, respectively. These two known sweet receptors function as G-protein coupled receptors that act via the IP3/cAMP pathway to release calcium from the endoplasmic reticulum, resulting in depolarization of the taste cell and release of neurotransmitter (21). While there are three known genes for the sweet receptor protein family, there are approximately 25 receptors that modulate bitter taste perception. The genes for bitter taste receptors (TAS2Rs) are clustered mainly on chromosomes 7 and 12 and wide variation exists in the perception of bitter taste. Differences in the abundance of mRNA in taste cells may cause variation in the number of receptors present on the papillae, which may explain the wide variation of bitter taste among those with the same genotype (22). As recently reviewed by D'Urso and Drago (23), TAS2Rs are capable of inducing bronchodilation and mucociliary clearance in the airways, muscle relaxation in various tissues, inhibition of thyroid stimulating hormone in thyrocytes and the release of glucagon-like peptide-1 (GLP-1_ and ghrelin in the digestive system. In contrast, TAS2Rs and taste 1 receptors (TAS1R2/3) are G protein-coupled receptors that are responsible for sweet taste perception that also may induce GLP-1 release an insulin secretion.

Genetic polymorphisms in taste receptors may contribute for some of the inter-individual differences in taste perception. For example, TAS2R38, a polymorphically expressed gene that modulates bitter taste sensation (14) has been widely studied for its role in food preferences, taste perception, immune responses, nutrition, and other pathophysiological mechanisms. This G-protein coupled receptor is responsible for the bitter taste of phenylthiocarbamide (PTC) and 6-*n*-propylthiouracil (PROP), thiourea compounds that serve as oral markers for individual differences in taste perception. There exist two common forms of the TAS2R38 protein due to single nucleotide polymorphisms (SNPs) within the gene coding for the TAS2R38 receptor. The individual differences in the tasting of PTC/PROP are determined by the TAS2R38 SNPs.

Teleologically, bitter taste perception was believed to protect humans from the intake of some poisonous substances during nutritional sustenance. Similarly, the ability to detect acidic taste can help maintain acid-base balance in the body by regulating the ingestion of acids. Similarly, the perception of salty taste facilitates discrimination against excessive sodium intake which facilitates homeostatic regulation of water balance, pH, conductance, and osmotic pressure (20).

Finally, as recently reviewed by D'Urso and Drago (23) it should be noted that extra-oral taste receptors exist in various organs and tissues such as the thyroid, lungs, skin, stomach, intestines, and pancreas. While their physiologic function is not yet fully understood, they may have pharmacologic significance in homeostasis regulation and host defense.

### Mouth feel—the role of viscosity on taste perception

The flavor of a given food and/or medication is only one factor when considering patient acceptance. Another important organoleptic property of taste is mouth feel, the oral sensations produced by a particular food which is dependent on many different characteristics (24). Viscosity, a rheological property of liquids, can also serve as a determinant of patient acceptance (25). It is defined as the measure of the resistance of a fluid to gradual deformation by shear or tension. In other words, viscosity describes the resistance of a fluid to flow. With regards to taste perception, properties such as the thickness, firmness, or ability of a food to break down are perceived first, followed by characteristics like creaminess or smoothness. As the size of individual particles in an oral suspension formulation of a drug grows smaller, the effective surface area of the particles increases and is associated with properties such as mouth after-feel and persistence of taste sensation. Together, these components combine to create a multidimensional texture that is influenced by the structure, rheology, and surface properties of a given ingestant (26). Because these factors are so closely intertwined, perceiving changes in a single property can be difficult. Increased viscosity has been shown to decrease the palatability of a liquid in both adults and children (24, 27). In a recent review by Chow, et al. (24), textural properties of food are particular drivers for food acceptance and rejection in children. As well, a child's acceptance of more complex food textures appears to be age-dependent and is influenced by repeated exposures to foods of differing geometrical textural properties (24). By inference, these findings could be easily extended to oral liquid drug formulations that are not true solutions (e.g., oral suspensions).

Attempts have been made to describe the relationship between the mouthfeel of liquid foods and their rheology (28). However, the ability of a liquid food to behave as a Newtonian fluid is directly impacted by factors such as saliva or deposition on the tongue or oral cavity. It is difficult to isolate certain attributes from one another, but it is generally believed that the perceived thickness of a fluid has the strongest link to viscosity. With low-viscosity liquids, a minimal amount of stress is needed to deform the fluid. On the other hand, viscous liquids require greater stress to be placed in order to maintain the rate of deformation (28). The thickness of the product can then be defined as how much the liquid resists the deformation, or the shear stress (28). A recent study has succeeded in determining how the perception of viscosity can vary (27).

On the human tongue, the viscosity of a liquid is detected by filiform papillae (21). Primarily located on the anterior two-thirds of the dorsal surface of the tongue, these papillae have mechanosensory endings that can transmit information to the brain in response to a viscous liquid. To be activated, fluids must have a high enough viscosity (a shear rate between 10 and 50 s^−1^) to deform the papillae. Individuals possessing longer, narrower filiform papillae in greater quantities are more adept at perceiving changes in viscosity (29). Additionally, previous data on age groups ranging from 21 to 84 have demonstrated a decrease in oral and oropharyngeal perceptions of fluid viscosity as age increases (30).

## Methods for assessing palatability of liquids in children

For nearly 3 decades, age-appropriate hedonic scales have been used to measure food preferences in young children (31) and have since been used to assess palatability of oral liquid medications in children. Previous “tools” that have been used to assess drug palatability in children include Likert scales, visual analogue scales, and facial recognition. Likert scales are responses within questionnaires ranging from 1 = very tasty to 5 = very bitter or 0 = disliked the taste to 4 = liked the taste. Likert scales are often used in conjunction with facial expressions to gain a better assessment of palatability in younger populations (32). Visual analogue scales allow the participant to select a point along a line to represent the degree of agreement with the statements written below the line, termed anchor phrases. The ends of the straight line are the extreme versions of the sensation being measured. In general, VAS shows good validity and sensitivity in children older than 7 years of age (33). In the past, facial recognition involved observation of the child by the researcher. If the child smiled while taking the medication, a “good taste” rating was given, no facial expression meant “acceptable,” and negative facial expressions or complaining meant “poor taste.” (34) More recently, parents were asked to rate palatability based on viewing their child's reaction or facial expression while being administered a medication as pleasant, unsure, or unpleasant (35). Overall, the 5-point visual analogue scale became the preferred method of testing palatability, but the conceptual ability of patients younger than 7 years to utilize it reliably was questioned.

In this younger age range (3–7 years of age), where reduced cognitive and neurodevelopmental statuses are normally present, other strategies such as facial recognition technology may have utility in an experimental setting (22). However, it should be noted that these technologies are not without their difficulties. Technical limitations with earlier approaches using facial recognition technology (13, 36) to assess palatability (taste preference and patient acceptance) include: requirement of sophisticated equipment and machine learning technology to record data and interpret the results; a significant amount of noise in the data output as multiple components of facial action/movement are recorded simultaneously and continuously to create a montage that is interpreted as a discrete result; the need for a controlled environment for facial recording that is not influenced by external/extraneous factors and the need for the child's caregiver to be involved in the evaluation so as to facilitate the accuracy of the recording (e.g., encouraging the child to not move and to look directly into the recording device).

**Figure 2:**
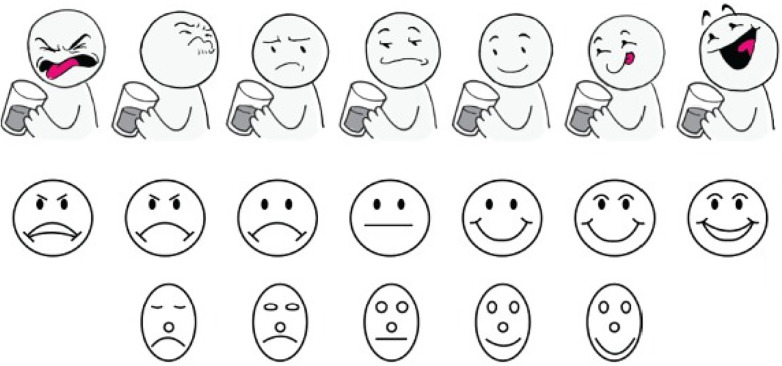
Seven-face and five-face study scales presented to subjects aged 5 years and older. Top to bottom: TASTY Scale (37), Chen Scale (31), Ellis Scale (38) (Chen and Ellis scales reproduced with permission).

The limitations seen with the aforementioned methods required the development of a more age-appropriate, patient-friendly, and reliable method to assess palatability (taste preference/patient acceptance), especially in very young children. To fill this gap, the TASTY scale has been newly created as a self-report taste rating scale to be utilized for palatability testing, particularly in younger children. The goal of creating this scale, in part, was to separate the perceptions of taste from emotion, which is commonly seen in existing faces scales (Figure 2) (31, 37, 38). Additionally, the TASTY scale was developed to be more child-friendly with the use of more graphically interesting images that included beverages and a body attached to the face. The assessment takes into consideration the neurodevelopmental status or stage of the child and their ability to aptly and correctly visually identify a response that they associate with their own response/reaction after receiving a given solution to taste. The response data are then transformed to a numeric value which enables testing of the impact of age as a developmental covariate with taste response. The 3-point version of the TASTY scale is intended for use with children 3 years old, the 5-point version for children 4 years old, and a 7-point scale for children ages 5 and older (37). When compared with two existing hedonic taste scales, the Chen (31) and Ellis (38) scales, the TASTY scale was preferred by both children and parents (37).

## Future directions

Although the Visual Analogue Scale (VAS) and facial hedonic scales continue to be widely used for the development and evaluation of liquid medications for children, they lack formal validation, are not appropriate for use in very young children (i.e., the population most likely to consume oral liquid medicines), and offer limited substantiation of the association between the palatability of medicines and pediatric adherence to treatment regimens (39). This dearth of standardized measurement tools introduces significant challenges in the comparison of results across different studies and impedes the development of a streamlined and uniform protocol for assessing medication palatability in the pediatric population. Based on the information accumulated to date, the scale with the greatest promise for use in pediatric drug development appears to be the Tasty Scale (37) as it is the most age-appropriate in terms of construction, ease of application, and reliability in the age ranges it has been studied. Further evaluation of this particular scale in very young children (e.g., those ages 2–5) is warranted.

Wide adoption of a single, standardized scale as the “gold standard” for assessing palatability of liquid drug formulations in infants and young children would enhance the reliability of outcomes across and between pediatric studies of palatability. Moreover, this would enable the relationship between medication palatability and pediatric adherence to be more robustly and conclusively tested. A unified, standard approach could potentially enhance the goals of pediatric drug therapy which, in turn, have the potential to improve health outcomes, mitigate therapeutic failures and lower health care costs. Most importantly, enhancing patient well-being improves the quality of life for the pediatric patient and their families.

## Conclusions

It is well recognized that the acceptability of a given medicine by a child is directly related to the drug formulation (40). While a standardized approach for creating uniform oral liquid drug formulations for different therapeutic categories is presently not attainable, a current opportunity does exist to standardize an approach (i.e., the TASTY scale) used to assess the palatability of oral drug formulations in children. While being “low tech,” this approach is widely available, designed especially for young children, is reproducible and highly reliable.

Finally, with pediatric drug development and regulation becoming increasingly a global exercise facilitated by harmonization of effort, the same goals are applicable regarding the tools/techniques used to assess palatability of drugs in pediatric patients. Realization of this goal has the potential to markedly improve pediatric drug development and thereby, the health of children everywhere.
